# Identification and phylogenetic analysis of seven *Brucella abortus* strains in Zhejiang, China

**DOI:** 10.3389/fcimb.2026.1758965

**Published:** 2026-05-22

**Authors:** Yong Yang, Xuguang Shi, Jiancai Chen, Lingbo Wang, Zhuoyin Wu, Wenwu Yao, Lizhi Xu, Yanjun Zhang, Zhiguo Liu, Beibei Wu

**Affiliations:** 1 Department of Microbiology, Zhejiang Center for Disease Control and Prevention (Zhejiang CDC), Hangzhou, China; 2 Department of Bioinformation, Beijing Macro&Micro-test Bio-Tech, Beijing, China; 3 National Key Laboratory of Intelligent Tracking and Forecasting for Infectious Diseases, National Institute for Communicable Disease Control and Prevention, Chinese Center for Disease Control and Prevention, Beijing, China

**Keywords:** ANI, *Brucella abortus*, brucellosis, cgSNP, pan-genome

## Abstract

**Introduction:**

*Brucella abortus* poses a serious zoonotic threat in China, however its genomic epidemiology in Zhejiang Province remains largely uncharacterized. This study addresses this knowledge gap by conducting the first integrated genomic analysis of seven *B. abortus* isolates obtained from patients and cows in the region.

**Methods:**

The phenotypic, genotypic, and phylogenomic characters of the isolates were illustrated by BCSP-31 and AMOS-PCR, MLST, ANI, cgSNP, and Pan-Genome analysis.

**Results:**

All seven isolates were identified as *B. abortus* via AMOS-PCR and belonged to a single sequence type (ST2) based on MLST, a classification further corroborated by an average nucleotide identity (ANI) of 99.99% to the reference strain *B. abortus* 544. Pan-genome analysis disclosed a highly conserved genome comprising 3,084 core genes and a minimal accessory genome of only 10 shell genes, alongside the prediction of 68 virulence-associated factors and one AMR gene, *mprF*. Core genome SNP phylogeny indicated that the Zhejiang strains form a monophyletic cluster with Russian references and trifurcate into three distinct subclades. Each subclade demonstrates specific phylogenetic connections to strains from separate northern Chinese provinces.

**Discussion:**

As the first study to integrate phenotypic, genotypic, and phylogenomic data for *B. abortus* in this region, our work substantially expands the understanding of its local population structure and provides a critical evidence base for proactive genomic surveillance, thereby informing targeted public health interventions against brucellosis.

## Introduction

*Brucella abortus* is a primary causative pathogen of bovine brucellosis, a disease that inflicts substantial economic losses on the global livestock industry (Mol et al., 2016). Its impact is characterized by abortion, infertility, and diminished milk production in cattle (Guimarães et al., 2019). As a zoonotic agent, *B. abortus* poses a direct threat to human health, causing irregular fever, arthritis, sweat and arthralgia, which can lead to severe chronic complications if untreated (de Figueiredo et al., 2015). Human brucellosis mainly spreads through the direct or indirect contact with infected livestock, consumption of contaminated raw dairy products and meat from domestic livestock (goats, sheep, cattle, pigs, camels and water buffalo) (Alamian and Dadar, 2019). *B. abortus* strains, distributed across 59 countries and regions spanning six continents, remain a significant public health risk for people in high-risk occupations (Liu et al., 2025). Human brucellosis caused by *B. abortus* are rare in southern provinces of China. From 2015 to 2025, only four *B. abortus* strains were isolated from patients in Zhejiang province, China. This scarcity stands in stark contrast to northern China and underscores an important gap in our full understanding of geographical distribution and regional transmission dynamics of the pathogens.

The advent of whole-genome sequencing (WGS) has revolutionized molecular epidemiology, and core-genome single nucleotide polymorphism (cgSNP) analysis now. It offers the highest resolution for investigating genetic relatedness and is particularly crucial for characterizing isolates and placing them accurately within the global phylogenetic context (Edao et al., 2023; Janke et al., 2023). Therefore, this study analyzes seven *B. abortus* strains from Zhejiang Province to determine their origins, and relationship to northern lineages, providing a basis for improved surveillance and control.

## Materials and methods

### Strain sources and identification

Seven *B. abortus* strains in this study, collected from the Zhejiang Province Brucellosis Surveillance Program between 2015 and 2025, consist of four patient-derived and three cow-derived isolates. The strains were stored at -40°C in a low-temperature freezer (Haier Biomedical, China) in the form of magnetic beads (Microbank, Miyagi Biomaterial Co., Ltd., Japan) in the laboratory of Zhejiang Provincial Center for Disease Control and Prevention. They were recovered and identified using standard bacteriological methods (Yagupsky et al., 2019). Briefly, the strains were streaked in sheep blood agar plates (Xinzhong, Shanghai, China), cultured at 37°C, 5% CO_2_ incubator (Heracell 240i, Thermo) for 3 days. Subsequently, the strains were re-identified via BCSP-31 and AMOS-PCR assays as previously described (Bankevich et al., 2012; Wingett and Andrews, 2018).

### Whole genome sequencing, assembly and pan-genome analysis

Genomic DNA of the strains were extracted and sequenced following the previously reported methods (Wang et al., 2025). Sequencing library preparation was performed using the Nextera XT library preparation kit (Illumina Inc., San Diego, CA, USA). After DNA quantification, fragmentation, end-repairing, A-tailing, Illumina adapter ligation, PCR amplification, and quality assessment, the DNAs were subjected to paired-end sequencing (PE150) on the Illumina NovaSeq X Plus platform. The raw sequencing reads quality assessment was conducted by FastQC (Wingett and Andrews, 2018), and assembly was performed using SPAdes (Bankevich et al., 2012). The Average Nucleotide Identity (ANI) was calculated using the Orthologous Average Nucleotide Identity Tool (OAT) (Lee et al., 2016). *In silico* multilocus sequence typing (MLST) was conducted on the assembled genomes using a previously described method (Vergnaud et al., 2018). Data visualization was performed using GrapeTree (v2.0) to generate a minimum spanning tree (MST) from MLST allelic profiles, applying the MSTree V2 (Edmonds’ algorithm) to reveal phylogenetic relationships (Zhou et al., 2018).

### Core-pan genome analysis and WGS-cgSNP

The core and pan-genome of seven *B. abortus* strains were analyzed with Panaroo (v1.2.10) under default parameters (Tonkin-Hill et al., 2020). Virulence-associated genes were predicted by screening the assembled genomes against the Virulence Factor Database (VFDB) using ABRicate (Chen et al., 2005). AMR-associated genes were detected through Resistance Gene Identifier (RGI) according to the ResFinder database (Zankari et al., 2012), and Comprehensive Antibiotic Resistance Database (CARD4.0.1) (Jia et al., 2016). For higher-resolution evolutionary analysis, a cgSNP-based phylogenetic tree was constructed. The genomic analysis pipeline began with the annotation of all study and global reference genomes using Prokka (Seemann, 2014). *B. abortus* 544 (GCA_000369945.1) was designed as the reference genome in this study. The core genome SNPs were called using Snippy v4.6.0, and putative recombinant regions were identified and filtered out using Gubbins v2.0.0 (Seemann, 2014). A maximum-likelihood phylogenetic tree was inferred from 551 SNP alignments including globally representative *B. abortus* and seven strains in this study using RAxML v8.2.13 (1,000 bootstrap replicates) (GTRGAMMA model) and visualized using iTOL v6 (Byndloss and Tsolis, 2016; Piao et al., 2018).

## Results

### Strains identified and genome sequence metrics of *Brucella abortus*

Colonies derived from human and cow on blood agar plates were smooth, convex, and translucent, matching the phenotypic profile of smooth *Brucella* strains. Using BCSP-31 and AMOS-PCR assays, all seven strains were identified as *B.abortus*, as evidenced by the amplification of specific 224 bp and 498 bp bands, respectively. These isolates were distributed in 3 cities (out of 11), most strains from Jinhua (n=4), followed by 2 in Quzhou and 1 in Ningbo (**Table 1**). The assembled draft genomes of the samples contained between 19 and 36 contigs longer than 500 bp, with total genome lengths ranging from 3,250,940 to 3,264,222 bp. The GC content of all isolates was between 57.24% and 57.26% (**Table 1**).

**Table 1:** Demographic and sequence metrics of seven *Brucella abortus* in this study.

Key	Species	ST	Host	Location	Year	Number of scaffolds	N50	GC content (%)
A1	*B.abortus*	2	cow	Jinhua	2018	24	383948	57.25
A2	*B.abortus*	2	cow	Jinhua	2018	25	383817	57.25
A3	*B.abortus*	2	cow	Jinhua	2018	24	383947	57.25
18BR22	*B.abortus*	2	human	Jinhua	2018	25	383930	57.25
22BR50	*B.abortus*	2	human	Ningbo	2022	36	384449	57.25
22BR56	*B.abortus*	2	human	Quzhou	2022	32	384404	57.26
22BR57	*B.abortus*	2	human	Quzhou	2022	19	384396	57.24

**Figure 1:**
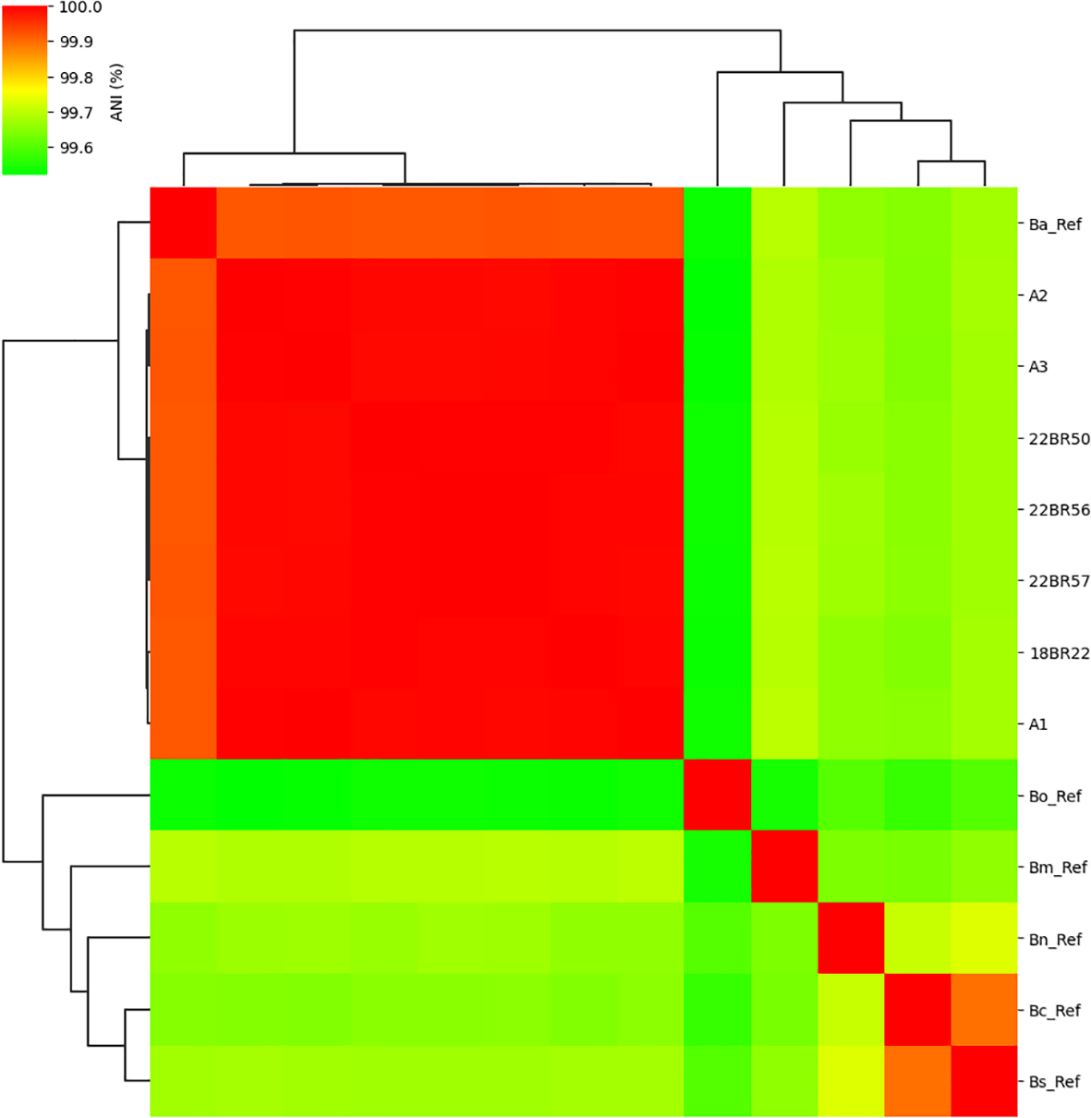
ANI of seven strains in this study to other six classical *Brucella* reference species. Ba_Ref, *B. abortus* 544; Bm_Ref, *B. melitensis* bv. 1 str. 16M; Bs_Ref, *B. suis* 1330; Bc_Ref, *B. canis* ATCC 23365; Bo_Ref, *B. ovis* ATCC 25840, and Bn_Ref, *B. neotomae* NCTC10084.

All samples exhibited relatively high assembly completeness, generally exceeding 99%. ANI analysis confirmed the strains as *B. abortus*, showing 99.99% identity to the reference strain *B. abortus* 544 (**Figure 1**). The number in the figure indicates the ANI values between two compared strains’ genomes.

### Pan-genome analysis profile of *Brucella abortus*

**Figure 2:**
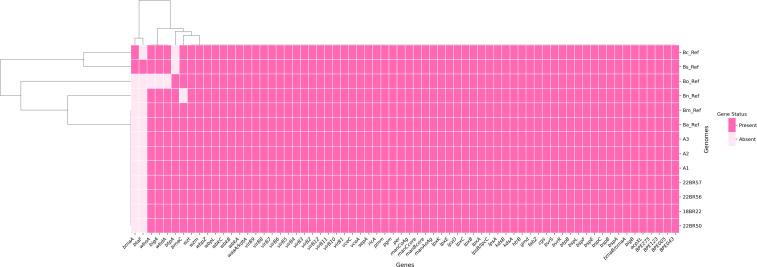
Displays the composition and distribution of virulence-associated genes in the seven strains (abricate 1.0.1; vfdb:2025-Nov-19; minimum DNA coverage & identity ≥ 80%).

Pan-genome analysis of the strains revealed a total of 3,094 genes. The genome structure is highly conserved, dominated by a core genome of 3,084 genes (present in ≥99% of strains). The accessory genome is notably small, comprising only 10 shell genes (present in 15% to <95% of strains), with no genes identified in the soft core (95% to <99%) or cloud (0% to <15%) categories. Unique genes per strain ranged from one to six. Furthermore, analysis of virulence genes identified 68 virulence-associated factors, included 30 LPS biosynthesis genes (VF0367), 15 T4SS-secreted effectors (VF0695), 12 VirB type IV secretion system components (VF0365), 5 Brucebactin synthesis genes (VF0692), 2 BvrR-BvrS regulatory genes (VF0368), and 4 additional virulence factors. The distribution pattern of these genes was largely consistent across all strains, with the *bmaA* and *btaF* genes being the exception, absent in all seven strains and *B. abortus* 544 (**Figure 2**). The *in silico* analysis of AMR genes in 7 genomes of *B. abortus* from Zhejiang, China, yielded only one multiple peptide resistance factor (*Brucella_suis*_*mprF*) in CARD and none in ResFinder.

### MLST analysis of *Brucella abortus*

The genetic relationship among the *Brucella* isolates was assessed through *in silico* multilocus sequence typing (MLST) based on nine housekeeping genes (*gap, aroA, glk, dnaK, gyrB, trpE, cobQ, int_hyp, omp25*). All seven *B. abortus* strains exhibited an identical allele profile (2, 1, 2, 2, 1, 3, 1, 1, 1), which corresponds to Sequence Type 2 (ST2), indicating genetic uniformity across the isolates. These findings are consistent with the results derived from whole-genome sequencing (WGS) data as referenced in public MLST databases.

### The core genome SNP phylogenetic analysis of *Brucella abortus* strains

**Figure 3:**
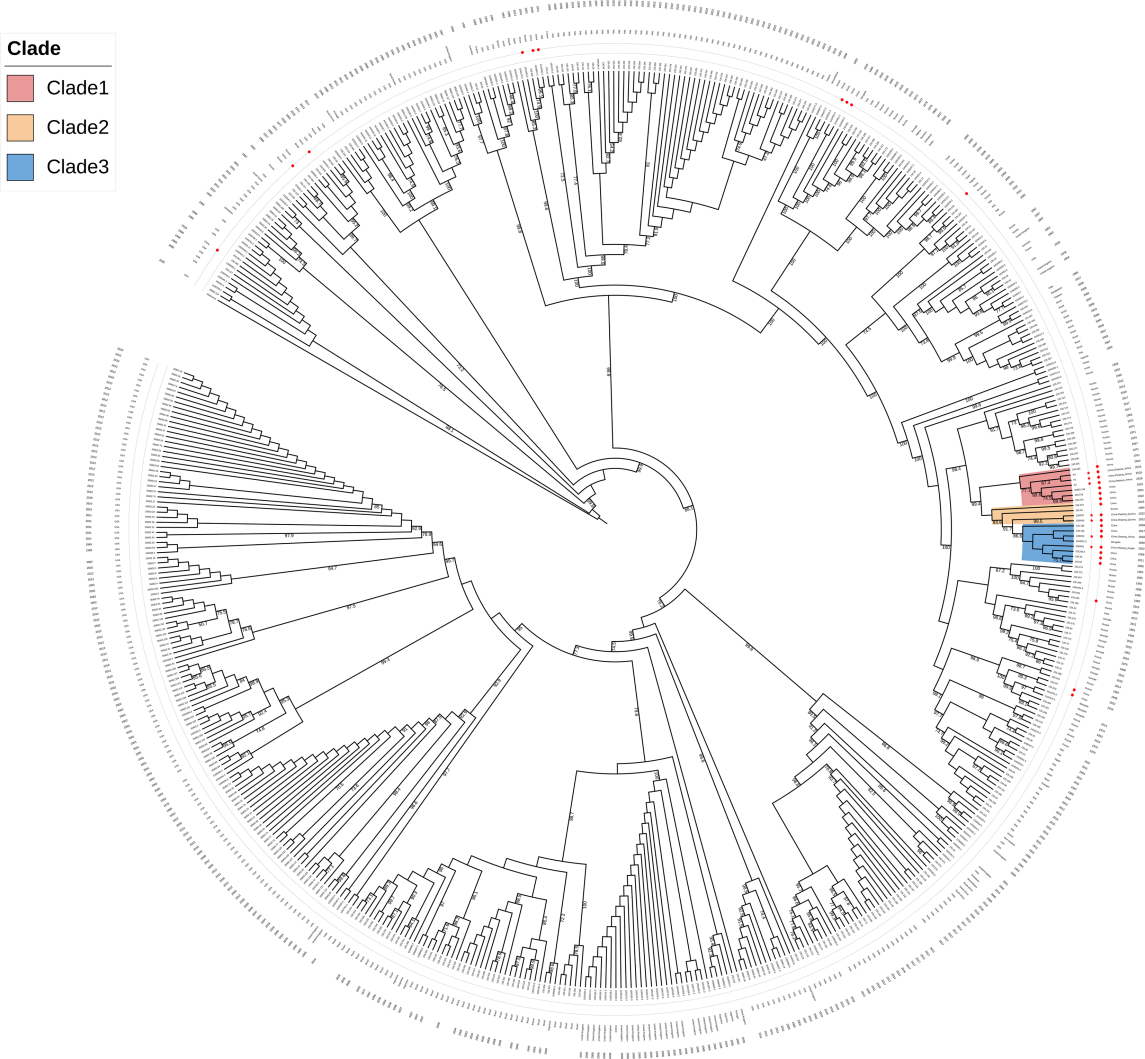
Whole-genome sequencing core-genome SNP (WGS-cgSNP) phylogenetic analysis of the seven *B. abortus* strains with 551 global *B. abortus* strains based on the Maximum-Likelihood algorithm with 1,000 bootstrap replicates.

The 10357 cgSNPs were called within the genomes of 551 *B. abortus* strains from the world. Based on a global phylogenetic analysis, 30 *B. abortus* strains from China were associated with the strains from Russia, Mongolia, USA, Brazil, India, Poland, and Spain, while the 7 isolates from Zhejiang province, China, were clustered closely with strains from Russia, Mongolia and the strains from northern provinces of China, indicating their placement within a narrower phylogenetic context (**Figure 3**).

**Figure 4:**
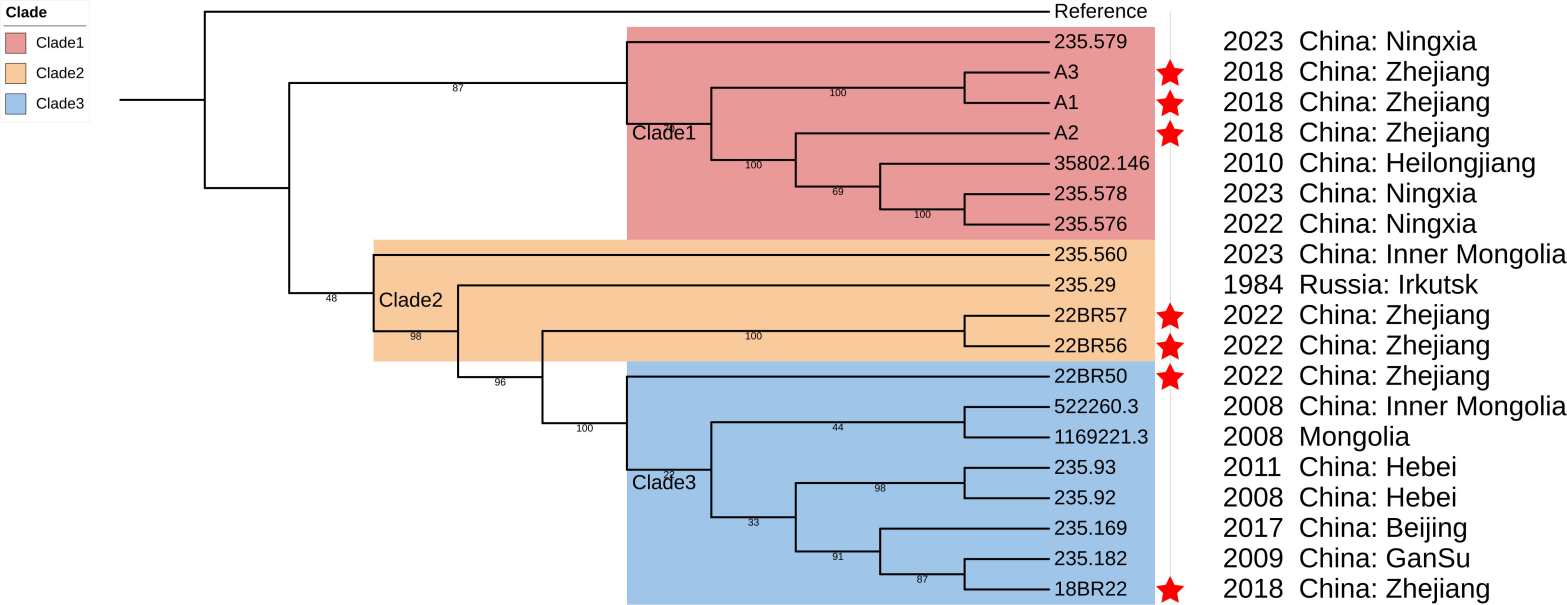
The sub-clades, containing seven strains from Zhejiang Province, China in **Figure 3**, were highlighted.

Within this major cluster, the Zhejiang strains separate into three clades that exhibit specific genetic relationships with isolates from other regions of China, Mongolia and Russia (**Figure 4**).

Clade 1, comprising strains A1, A2, and A3 from Zhejiang, shows phylogenetic affinity with isolates from Ningxia and Heilongjiang provinces. Clade 2, consisting of two Zhejiang strains, demonstrates genetic relatedness to isolates from Inner Mongolia and Russia. Clade 3, composing of another two Zhejiang strains, indicates evolutionary proximity to isolates from Mongolia, Inner Mongolia, Hebei, Gansu and Beijing. The observed clustering patterns suggest possible pathogen flow among these regions, though the direction and mechanisms of such potential spread would benefit from further investigation with enhanced epidemiological data.

## Discussion

This study presents the first integrated molecular characterization of *B. abortus* in Zhejiang Province, combining bacteriological methods, AMOS-PCR, MLST, pan-genome and cgSNP phylogenetic analysis. The successful isolation and identification of *B. abortus* from both human and cow significantly expands the known pathogen spectrum in this region, providing critical evidence for strengthening brucellosis surveillance at the human-animal interface. From a public health perspective, this finding underscores the ongoing zoonotic transmission of *B. abortus* and highlights the necessity of strengthening joint human-animal health surveillance. MLST analysis demonstrated that all seven strains belong to ST2, indicating local circulation of a clonal lineage. Pan-genome analysis further revealed a highly conserved genome with a large core gene set (3,084 genes) and minimal accessory genes (10 shell genes), reflecting genetic stability and low horizontal gene transfer activity. These results imply that conventional genotyping tools such as MLST remain effective for tracking and monitoring *B. abortus* in this region (Piao et al., 2018).

Virulence factor screening identified 68 associated genes, most of which were conserved across all isolates. The *bmaA* codes for the autotransporter outer membrane beta-barrel domain-containing protein BmaA (VF1339) (Bialer et al., 2021). The *btaF, Brucella* TAD adhesin F, encodes a critical outer membrane protein that serves as a major pilus subunit and adhesin (Byndloss and Tsolis, 2016; Bialer et al., 2020). Their biological significance is profound, as they are key players in the bacterial ability to establish and maintain successful chronic infections in hosts. However, the absence of the *bmaA* and *btaF* genes in seven strains and *B. abortus* 544 suggests possible variation in animal host adaptation or pathogenic strategies (Bialer et al., 2020; Suárez-Esquivel et al., 2020). Although the functional impact requires further study, this finding highlights the value of incorporating virulence gene annotation into genomic surveillance. The analysis of WGS data of 7 Zhejiang isolates revealed one AMR gene, multiple peptide resistance factor (*mprF*), which plays an essential role in resistance to cationic antibiotics such as gentamicin, moenomycin, and vancomycin in *Staphylococcus aureus* (Nishi et al., 2004). None of our 7 isolates exhibited resistance to the gentamicin. Although the genetic determinants of antibiotic resistance in *B. abortus* were investigated, the specific role of *mprF* in antimicrobial resistance in *Brucella* remains unclear (Sandalakis et al., 2012; Yang et al., 2022; Abdallah et al., 2025).

The cgSNP phylogeny provided the highest resolution for confirming the genetic relatedness and precise phylogenetic placement of these rare Zhejiang strains within the global *B. abortus* population, which is essential for tracing their origins (Dadar et al., 2023). These findings collectively suggest that while the Zhejiang strains may share evolutionary background with Russian isolates, they appear to have diversified into multiple lineages with connections to different regional populations.

The wgSNP-based phylogenetic placement of 46 Kazakhstan strains indicates significant genetic relatedness to those from geographically proximate areas, notably North Caucasia and Western Russia, with additional links to Siberia, China, and Mongolia (Shevtsov et al., 2024). The phylogenetic patterns observed provide insights into the potential transmission dynamics of *B. abortus* in the region, though further investigation would be valuable to fully elucidate the underlying epidemiological relationships. Furthermore, cgSNP analysis resolved the Zhejiang strains into three subclades with specific genetic links to those from Russian, Mongolia and multiple northern Chinese provinces. The phylogenetic placement of strain BD002 from Tianjin, which formed a distinct sub-clade with strains from multiple North Chinese regions and neighboring countries, suggests dissented from a common lineage (Gao et al., 2025). The phylogenetic analysis revealed that the 12 *B. abortus* strains from Shandong formed genetic connections with strains from Heilongjiang, Inner Mongolia, Mongolia, and Russia (Huangfu et al., 2025). These data implicating broader transmission dynamics than previously recognized. This high-resolution phylogeny not only revealed previously unrecognized transmission chains but also highlighted the existence of a disseminated clade that transcends national and provincial borders. The granular insight from cgSNP data is critical for enabling precision public health, as it allows for targeted interventions including movement control and risk-based screening by pinpointing introduction sources and clarifying transmission dynamics.

## Conclusion

Based on an integrated approach combining bacteriology, MLST, pan-genome and cgSNP phylogenetic analysis, this study provides the first high-resolution molecular characterization of *B. abortus* in Zhejiang Province. We revealed that while local strains exhibit a clonal structure (ST2) by MLST, they display fine-scale phylogenetic diversity through cgSNP profiling, with clear genetic links to external sources. These findings refine the understanding of the pathogen’s local genetic background and potential transmission patterns, while offering critical evidence for modernizing brucellosis surveillance. Future studies should integrate epidemiological data to elucidate the drivers of external introductions and implement extensive genomic surveillance to track real-time transmission dynamics.

## Data Availability

The datasets presented in this study can be found in online repositories. The names of the repository/repositories and accession number(s) can be found below: https://nmdc.cn/submit/mysubmission, NMDC40061828.
